# HIV Symptom Burden and Anemia among HIV-Positive Individuals: Cross-Sectional Results of a Community-Based Positive Living with HIV (POLH) Study in Nepal

**DOI:** 10.1371/journal.pone.0116263

**Published:** 2014-12-31

**Authors:** Catherine Martin, Kalpana Poudel-Tandukar, Krishna C. Poudel

**Affiliations:** Department of Public Health, School of Public Health and Health Sciences, University of Massachusetts Amherst, Amherst, MA, United States of America; Alberta Provincial Laboratory for Public Health / University of Alberta, Canada

## Abstract

**Background:**

Previous research has reported high rates of anemia in people living with HIV/AIDS (PLWHA) in hospital or tertiary care settings. The objective of this community-based study was to measure the prevalence of anemia and describe the risk factors, with a specific emphasis on HIV symptom burden, in PLWHA in the Kathmandu Valley, Nepal.

**Methods:**

We conducted a cross-sectional survey of 319 PLWHA residing in the Kathmandu Valley, Nepal. We recruited participants from five non-governmental organizations in the Kathmandu Valley. Descriptive statistics and multivariable logistic regression analyses were used.

**Results:**

Our study found a 55.8% prevalence of anemia in PLWHA in the Kathmandu Valley. The prevalence of anemia among the participants with first, second, third, and fourth quartiles of HIV symptom burden was 44.8%, 49.3%, 60.3%, and 69.6%, respectively. Compared to the participants with lowest level of HIV symptom burden, the participants with highest level of HIV symptom burden were more likely to have anemia (adjusted odds ratio = 2.14; 95% confidence interval = 1.07 to 4.30).

**Conclusion:**

Due to a high prevalence of anemia in a community-based sample of PLWHA, HIV patients should be counseled on their risk of developing anemia and encouraged to seek timely care for HIV symptoms.

## Introduction

Anemia is an important contributor to morbidity and mortality among people living with HIV/AIDS (PLWHA). In different study settings, the prevalence of anemia in patients with clinical AIDS has been estimated between 63% and 95% [1], [2], [3], making it the most common hematologic manifestation of the disease [1], [2], [4], [5]. Even among people living with HIV but without clinical AIDS, estimates of anemia are high and range from 18%–82.7% [3], [4], [6], [7].

Although comparing rates of anemia among PLWHA from different studies is complicated by variations in a number of factors including stage of HIV disease, use of ART, and definition of anemia, results from a review of the literature suggest that rates of anemia in PLWHA in a number of subpopulations tend to be higher in developing countries than in developed countries [4]. For example, the prevalence of anemia (defined as hemoglobin level <110 g/L) in HIV-positive pregnant women in Tanzania [8], Burkina Faso [9], and the Ivory Coast [10] was 82.7%, 78.4%, and 81.7%, respectively while the prevalence of anemia (defined as hemoglobin levels <100 g/L) in HIV-positive pregnant women in Italy [11] was 27.2%. Among non-pregnant HIV-infected women in varying stages of disease progression, anemia prevalence in the United States [12], [13] (defined as hemoglobin levels <120 g/L) ranged from 24.8% to 44.9%, while in Iran, 72% of non-pregnant HIV-infected women were anemic (defined as hemoglobin <120 g/dL) [1]. Similarly, among HIV-positive injection drug users in Malaysia the prevalence of anemia was 81.6%, while rates in the USA and the Netherlands (defined as 130 g/L for men and <120 g/L for women) were between 21.1% and 46.1% [12], [14], [15], [16]. Thus, studies suggest that PLWHA in developing countries are especially vulnerable to anemia.

Anemia can have severe consequences for PLWHA. Anemia can cause fatigue, shortness of breath, and increased heart rate, all of which substantially worsen a patient's quality of life [5], [17]. Moreover, anemia can impact HIV disease progression, treatment options, and mortality. Several studies [18], [19], [20] have indicated that among individuals with HIV, anemia is associated with HIV progression to AIDS. Furthermore, HIV patients with anemia are at a greater risk of mortality compared to their non-anemic counterparts, even after controlling for CD4+ cell count and viral load [4], [21].

HIV disease and treatment are believed to result in decreased red blood cell (RBC) production, increased RBC destruction, and/or ineffective RBC production, thus increasing the prevalence of anemia among PLWHA [1], [5]. Several factors have been identified that put PLWHA at an increased risk of developing anemia. These are later stage of HIV disease [2], [3], CD4+ cell count of less than 200 cells/µL [3], [13], [21], increased viral load [13], [21], female sex [1], [3], [21], [22], [23], being pregnant [4], [10], injection drug use [4], [14], and use of Zidovudine and Ganciclovir [3]. In developing countries, lower levels of education [30], poorer housing [30], rural residence [31], unemployment [30], poor diet [30], lower BMI [28], increased age [2], and presence of other infections [28] have also been associated with increased levels of anemia.

To the best of our knowledge, the current study is the first to describe the prevalence of anemia and associated risk factors, with a specific emphasis on HIV symptom burden, using a community-based sample of HIV-positive people in a developing country context in Asia. Previous studies assessing anemia rates among PLWHA in Asian countries such as India [22], [24], [25], [26], China [2], and Iran [27] have found rates between 41%–75%. However, these studies either recruited participants from a hospital or tertiary care settings [22], [24], [25], [26], [27] or recruited only newly diagnosed HIV patients who had not received ART [2]. The results of these studies, therefore, may not be generalizable to the larger population at various stages of disease progression and with varying access to tertiary care facilities.

The objective of this community-based study was to measure the prevalence of anemia and describe the risk factors, with a specific emphasis on HIV symptom burden, in PLWHA in the Kathmandu Valley, Nepal, a resource-limited country in south Asia. A study in India found that symptomatic HIV-positive participants were more likely to be anemic than non-symptomatic participants [24]. However, the study defined being symptomatic as having a single symptom. The current study hopes to more thoroughly describe the relationship between HIV symptom burden and anemia prevalence. Individuals with a higher burden of HIV symptoms may have a higher prevalence of anemia because increased symptom burden may represent more opportunistic infections and increased inflammation, both of which are connected with anemia [28], [29]. Since anemia so adversely affects the prognosis of HIV, it is important to characterize the prevalence and risk factors of anemia in PLWHA in Nepal. Doing so will help identify the patients at greatest risk for adverse effects and allow health care providers to better address anemia among this population and ultimately reduce the negative effects of anemia and improve patients' quality of life.

## Methods

### Study Area

The study was conducted in the Kathmandu Valley, Nepal. The Kathmandu Valley consists of three districts: Kathmandu, Bhaktapur, and Lalitpur and in 2011 had an estimated population of about 2.5 million people. Located in the Hills region of Nepal, the Kathmandu Valley has an approximate elevation of 1400 meters. Of 39 ART sites in Nepal, six are located in the Kathmandu Valley.

As of 2011, Nepal was categorized as having a concentrated HIV epidemic with an estimated number of 50,200 adults and children with HIV [30]. Although only about 0.3% of the general population is infected with HIV infection in Nepal, the rates are much higher in people who inject drugs (6.3%), female sex workers (4.2%), men who have sex with men (3.8%), and male labor migrants (1.8%) [30], in particular to Mumbai [31]. Despite the introduction of free ART services in 2004, coverage to eligible patients was just 24% in 2011 in Nepal [30]. Of those, approximately 80% were receiving a combination of drugs that include Zidovudine [32]. Anemia is also an important health concern in Nepal. The World Health Organization (WHO) estimates that, worldwide 24.8% of people are affected by anemia [33]. Nepal, however, experiences significantly higher rates of anemia. In 1998, the Nepal Micronutrient Status Survey estimated rates of anemia to be as high as 66% in non-pregnant women [34]. More recently, the 2011 Demographic Health Survey estimated rates of anemia among non-pregnant women to be 34.8% [35] indicating the levels of anemia in Nepal have improved in the last decade but remain a serious health concern. In the Kathmandu Valley, there is a high rate (31.1%) of intestinal parasitic infections [36], one of the causes of anemia. Malaria and schistosomiasis are rare in the Kathmandu Valley [37], [38].

Overall, Nepal has several characteristics in common with other developing countries in Asia such as low HIV prevalence in the general population but high in sub- populations considered as high-risk groups for HIV infection and who have low rates of ART coverage [39].

### Study Participants

Between February and March of 2010, we collected data on 322 participants as part of a baseline survey for a longitudinal healthy living study entitled “Positive Living with HIV” (POLH) headed by the last author [40], [41], [42], [43], [44]. Staff from five non-governmental organizations that worked with HIV-positive people in the Kathmandu Valley recruited participants. In order to be eligible for the study, participants needed to be at least 18 years of age, self-report an HIV-diagnosis, and give written informed consent voluntarily.

### Procedures

We used a structured questionnaire for data collection. We developed the questionnaire in English, translated it to Nepali, and then back-translated it to English. The Nepali version was revised after back-translation, pre-tested on 30 HIV-positive people, and modified a final time to reflect the pre-test results.

Four interviewers collected data by face-to-face interview. First, these interviewers received a one-day training regarding the contents of the questionnaire and interview technique. Then they conducted the in-person interviews individually in a private setting using the structured questionnaire. Each interview took approximately 45 minutes. Participants were reimbursed 100 Nepali rupees (approximately US $1.35) for transportation costs. The second and third authors supervised the fieldwork.

After the interview, laboratory technicians collected approximately 10 mL of venous blood from each participant. After 5 to 15 minutes, they centrifuged the blood for 15 minutes and separated the serum. A hemoglobin (Hb) concentration <13.0 g/dl (mild = 11.00–12.0 g/dl; moderate = 8.00–10.9 g/dl; and severe = <8.0 g/dl) in men and <12.0 g/dl (mild = 11.00–11.9 g/dl; moderate = 8.00–10.9 g/dl; and severe = <8.0 g/dl) in non-pregnant women is defined as anemia [33]. The prevalence of anemia among people living at high altitude may be underestimated when standard cutoffs apply as residential elevation of over 1000 meters above sea level increases Hb concentrations. As WHO recommends [33], we adjusted 0.3 g/dl in our cutoff values as the Kathmandu Valley, our study area, is located about 1400 meters above sea level.

### Measures

Socio-demographic measures included age, sex, current marital status, education, and employment status. Health characteristic measures included months since HIV-diagnosis, ART status, current smoking status, lifetime injection drug use, alcohol consumption, and HIV symptom burden. These variables were measured adopting the questionnaire from previous studies conducted in Nepal [31], [45], [46], [47].

We characterized participants as “current smokers” if they reported smoking “every day” or “some days.” [48] We assessed lifetime IDU by asking whether the participants had ever used drugs by injection. We assessed alcohol consumption by asking participants if they had consumed alcohol in the past 30 days.

We measured HIV symptom burden using a 16-item HIV-symptom index questionnaire [49]; the Cronbach α for this scale was 0.92 in this study. Participants selected their answer on a five-option response scale, which ranged from 0 (*I don't have this problem*) to 4 (*It bothers me a lot*). Selection was based on a 1-month recall period. The score of 16 items was combined to create an overall index of HIV symptom burden. The overall index of HIV symptom burden had a range of 16 (lowest level of HIV symptom burden) to 80 (highest level). The median and inter-quartile range (IQR) of the overall index of HIV symptom burden in our study was 32.0 and 24 to 45, respectively.

### Data Analysis

Of the 322 participants, three were excluded from the data analysis because they did not provide a blood sample; thus we included the responses of 319 participants in our data analysis. For analysis, first, we calculated descriptive statistics for socio-demographic and other relevant characteristics. We reported mean and standard deviation (SD) or median and IQR as appropriate. Second, we compared the participants' characteristics according to quartile of HIV symptom burden. Third, we examined the bivariate associations between each independent variable and anemia. We obtained unadjusted odds ratio (OR) and 95% confidence interval (CI) using logistic regression analysis. Finally, we examined the association between HIV symptom burden and anemia using multivariable logistic regression analysis including all the variables that had a bi-variate p-value less than or equal to 0.20 or those variables that were associated with anemia in previous studies, such as age. Our final multivariable model adjusted for age, sex, education, months since testing HIV-positive, ART status, history of injecting drug use, and HIV symptom burden. We performed all of the analyses using SPSS version 15.0 (SPSS Inc., Chicago, USA) for windows with statistical significance set at p<0.05.

### Ethical Considerations

The procedures of the study were reviewed and approved by the Research Ethics Committee of the Nepal Health Research Council in Kathmandu, Nepal; National Center for Global Health and Medicine in Tokyo, Japan; Waseda University in Tokyo, Japan, and institutional review board of the University of Massachusetts Amherst. Participation in the study was voluntary and confidential. Prior to each interview, the interviewers informed the participant of the study procedures and reassured participants that codes would replace names and other identifying information to maintain confidentiality. All participants were required to sign an informed consent form before taking part in the study.

## Results

### General Characteristics

The mean age of 319 participants was 35.6 years (SD = 6.9 years). The median duration since testing positive for HIV was 53 months (IQR = 25–86). Of total, 57.4% of participants were men, 68.7% were currently married, 31.7% were unemployed, 73.1% were receiving ART, 40.8% reported lifetime injection drug use, and 49.2% had high levels of HIV symptom burden (over 32 on the index of HIV symptom burden). Of the 233 participants on ART, 226 (96.9%) were under two Nucleoside/Nucleotide Reverse Transcriptase Inhibitors (NRTIs; any two of Lamivudine, Stavudine, and Zidovudine) and one Non-Nucleoside Reverse Transcriptase Inhibitors (NNRTIs; either Efavirenz or Nevirapine), 6 (2.6%) were under two NRTIs (Didanosine and Tenofovir) and one Protease Inhibitor (PI; Lopinavir/Ritonavir), and one participant was under three NRTIs (Abacavir, Lamivudine, and Zidovudine). In total, 178 (76.4%) were taking a zidovudine-containing ART.

Characteristics of participants according to quartile of HIV symptom burden are shown in Table 1. A much higher proportion of participants aged between 20 and 34 years old (62.0%) reported highest levels of HIV symptom burden compared to those aged between 35 and 60 years old (38.0%). Similarly, a much higher proportion of female participants (59.5%) reported highest levels of HIV symptom burden compared to those of male participants (40.5%). A much smaller proportion of participants with up to primary level education (20.7%) reported lower levels of HIV symptom burden than those with secondary or higher levels of education (79.3%). A higher proportion of participants with fewer months (1–35 months) since testing HIV positive (40.5%) reported highest levels of HIV symptom burden than those with more months (73–258 months) since testing HIV positive (27.8%).

**Table 1:** Socio-demographic and health characteristic of participants according to quartile of HIV symptom burden (n = 319).

Variables	HIV Symptom Burden								*P* value
	Q1 (Lowest)		Q2		Q3		Q4 (Highest)		
	(n = 87)		(n = 75)		(n = 78)		(n = 79)		
	n	(%)	n	(%)	n	(%)	n	(%)	
**Age (in years)**									
20–34	48	(55.2)	50	(66.7)	35	(44.9)	49	(62.0)	0.037
35–60	39	(44.8)	25	(33.3)	43	(55.1)	30	(38.0)	
**Sex**									
Female	20	(23.0)	32	(42.7)	37	(47.4)	47	(59.5)	<0.001
Male	67	(77.0)	43	(57.3)	41	(52.6)	32	(40.5)	
**Current marital status**									
Single	21	(24.1)	25	(33.3)	27	(34.6)	27	(34.2)	0.404
Married	66	(75.9)	50	(66.7)	51	(65.4)	52	(65.8)	
Education^a^									
Up to primary	18	(20.7)	34	(45.9)	33	(42.9)	43	(54.4)	<0.001
Secondary or higher	69	(79.3)	40	(54.1)	44	(57.1)	36	(45.6)	
**Employed**									
No	21	(24.1)	26	(34.7)	23	(29.5)	31	(39.2)	0.183
Yes	66	(75.9)	49	(66.3)	55	(70.5)	48	(60.8)	
**Months since testing HIV positive**									
Tertile 1 (1–35)	18	(20.7)	25	(33.3)	31	(39.7)	32	(40.5)	0.019
Tertile 2 (36–72)	37	(42.5)	19	(25.3)	29	(37.2)	25	(31.6)	
Tertile 3 (73+)	32	(36.8)	31	(41.3)	18	(23.1)	22	(27.8)	
**On antiretroviral therapy**									
No	25	(28.7)	20	(26.7)	20	(25.6)	21	(26.6)	0.357
Zidovudine not-containing	11	(12.6)	14	(18.7)	10	(12.8)	20	(25.3)	
Zidovudine containing	51	(58.6)	41	(54.7)	48	(61.5)	38	(48.1)	
**Lifetime injection drug use**									
No	45	(51.7)	47	(62.7)	46	(59.0)	51	(64.6)	0.344
Yes	42	(48.3)	28	(37.3)	32	(41.0)	28	(35.4)	
**Current smoker**									
No	42	(48.3)	40	(53.3)	41	(52.6)	46	(58.2)	0.647
Yes	45	(51.7)	35	(46.7)	37	(47.4)	33	(41.8)	
**Alcohol use in past 30 days**									
No	75	(86.2)	63	(84.0)	69	(88.5)	72	(91.1)	0.575
Yes	12	(13.8)	12	(16.0)	9	(11.5)	7	(8.9)	

a Two participants did not respond to this question.

### Prevalence of Anemia

**Figure 1:**
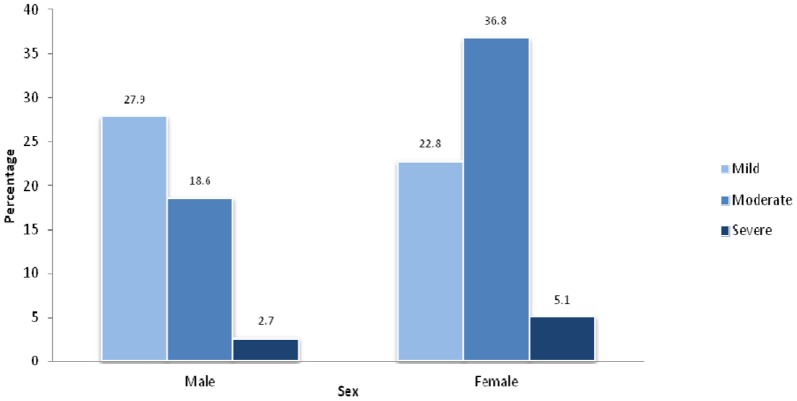
Prevalence of anemia among male and female HIV-positive people in the Kathmandu Valley, Nepal.

A total of 178 participants (55.8%) were anemic. The prevalence of mild, moderate, and severe anemia among the participants was 25.7%, 26.3%, and 3.8%, respectively. The prevalence of anemia among female participants was 64.7% while it was 49.2% among male participants. Among males, the prevalence of mild, moderate, and severe anemia was 27.9%, 18.6%, and 2.7%, respectively (**Fig. 1**). Among females, the prevalence of mild, moderate, and severe anemia was 22.8%, 36.8%, and 5.1%, respectively.

**Figure 2:**
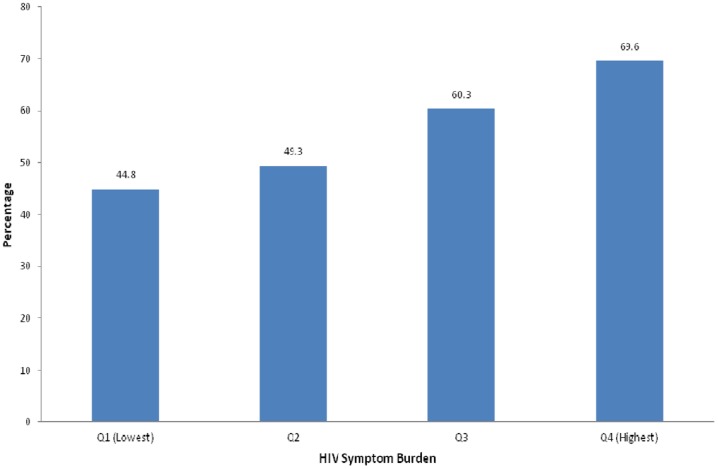
Prevalence of anemia according to the HIV symptom burden among HIV-positive people in the Kathmandu Valley, Nepal.

The prevalence of anemia increased among the participants with the increase of their HIV symptom burden (**Fig. 2**). For example, the prevalence of anemia among the participants with first, second, third, and fourth quartiles of HIV symptom burden was 44.8%, 49.3%, 60.3%, and 69.6%, respectively.

### Factors Associated with Anemia in HIV Patients

Factors associated with anemia in HIV-positive participants in the bivariate analysis are shown in Table 2. In the multivariable analysis, HIV symptom burden was statistically associated with the prevalence of anemia among HIV-positive participants in the Kathmandu Valley after adjusting age, sex, education, months since testing HIV-positive, and ART status. Compared to the participants with lowest levels of HIV symptom burden, the participants with highest level of HIV symptom burden were 2.14 times more likely to have anemia (AOR = 2.14; 95% CI = 1.07–4.30). There was no statistical interaction of HIV symptom burden and months since testing HIV-positive (p = 0.343). Similarly, the interaction between HIV symptom burden, age, and months since testing HIV-positive was not statistically significant (p = 0.581).

**Table 2:** Factors associated with anemia among HIV-positive people in the Kathmandu Valley, Nepal.

Variables	Anemia				OR	(95% CI)	AOR^a^	(95% CI)
	Yes		No					
	n	(%)	n	(%)				
**Age (in years)**								
20–34	103	(56.6)	79	(43.4)				
35–60	75	(54.7)	62	(45.3)	0.93	(0.59–1.45)	0.89	(0.54–1.48)
**Sex**								
Female	88	(64.7)	48	(35.3)				
Male	90	(49.2)	93	(50.8)	0.52	(0.33–0.83)	0.79	(0.41–1.53)
**Current marital status**								
Single	52	(52.0)	48	(48.0)				
Married	126	(57.6)	93	(42.5)	1.25	(0.77–2.01)		-
**Education** b								
Up to primary	85	(66.4)	43	(33.6)				
Secondary or higher	91	(48.1)	98	(51.9)	0.47	(0.29–0.74)	0.64	(0.37–1.08)
**Employed**								
No	60	(59.4)	41	(40.6)				
Yes	118	(54.1)	100	(45.9)	0.80	(0.50–1.30)		-
**Months since testing HIV positive**								
Tertile 1 (1–35)	69	(65.1)	37	(34.9)				
Tertile 2 (36–72)	59	(53.6)	51	(46.4)	0.62	(0.35–1.07)	0.81	(0.45–1.46)
Tertile 3 (73+)	50	(48.5)	53	(51.5)	0.50	(0.29–0.88)	0.61	(0.33–1.11)
**On antiretroviral therapy**								
No	39	(45.3)	47	(54.7)				
Zidovudine not-containing	32	(58.2)	23	(41.8)	1.67	(0.84–3.32)	1.54	(0.73–3.25)
Zidovudine containing	107	(60.1)	71	(39.9)	1.81	(1.08–3.05)	1.68	(0.95–2.96)
**Injection drug use in past 6 months**								
No	114	(60.3)	75	(39.7)				
Yes	64	(49.2)	66	(50.8)	0.63	(0.40–1.00)	0.96	(0.51–1.83)
**Current smoker**								
No	99	(58.6)	70	(41.4)				
Yes	79	(52.7)	71	(47.3)	0.78	(0.50–1.22)		-
**Alcohol use in past 30 days**								
No	158	(56.6)	121	(43.4)				
Yes	20	(50.0)	20	(50.0)	0.76	(0.39–1.48)		-
**HIV Symptom burden**								
Quartile 1 (Lowest)	39	(44.8)	48	(55.2)				
Quartile 2	37	(49.3)	38	(50.7)	1.19	(0.64–2.22)	0.96	(0.49–1.85)
Quartile 3	47	(60.3)	31	(39.7)	1.87	(1.00–3.46)	1.48	(0.76–2.88)
Quartile 4 (Highest)	55	(69.6)	24	(30.4)	2.82	(1.45–5.34)	2.14	(1.07–4.30)

Abbreviations: OR, odds ratio; CI, confidence interval; AOR, adjusted odds ratio.

a A total of 317 participants were included in the multiple regression analysis.

b Two participants did not respond to this questions.

## Discussion

To our knowledge, this is the first community-based study measuring the prevalence of anemia and associated risk factors, with particular emphasis on HIV symptom burden, in HIV-positive people in a developing country context. This study reported a 55.8% prevalence of anemia among HIV-positive people in the Kathmandu Valley, Nepal. Anemia was more common among participants who reported more HIV symptoms.

The prevalence of anemia in HIV-positive people in this study was similar to rates reported from hospitals or tertiary care settings in other Asian countries such as India (41%–74.4%) [22], [24], [25], [26], Iran (46%) [27], and China (51.9%) [2]. A number of factors might have contributed to high rates of anemia among the HIV-infected participants in this study. These factors may include multiple other infections as our study participants with higher HIV symptom burden have higher prevalence of anemia; infrequent monitoring for anemia, resulting in missed opportunities for treatment; nutritional deficiencies, and parasitic infections as the prevalence of hookworm and other soil-transmitted helminth infection is high in the Kathmandu Valley [36].

Our results indicate that HIV symptom burden is positively correlated with prevalence of anemia. In a study in India [24], the prevalence of anemia was significantly higher among HIV-positive participants who had one or more HIV symptoms. Our results indicate that the prevalence of anemia increases as the reported number of HIV symptoms increases, with participants reporting the highest burden of symptoms about two times more likely to be anemic compared to those reporting the lowest burden. An increased burden of HIV symptoms may represent the presence of opportunistic infections and/or increased inflammation, which both can contribute to the development of anemia. Inflammation can cause anemia in the general population and thus anemia is often observed in patients with acute or chronic infections [28]. Moreover, many opportunistic infections common in HIV infection have been connected to anemia [29], [50].

Several reports indicate an increasing prevalence of anemia as HIV disease progresses [3], [4], [21], [51]. These studies measured disease progression as a function of CD4 count, viral load, or the development of opportunistic infection, where a CD4+ cell count of less than 200 cells/µL [3], [13], [21], increased viral load [13], [21], and opportunistic infections such as tuberculosis, candidiasis, lymphadenopathy, and chronic diarrhea [22] are associated with an increased prevalence of anemia. It is likely that individuals in later stages of disease progression, as measured by CD4+ count, viral load, or opportunistic infections, also experience a higher burden of HIV symptoms. Thus, these factors may have an additive effect in the development of anemia among PLWHA. Therefore, addressing symptoms associated with HIV/AIDS might help to reduce the burden of anemia in this population. Health care workers should ensure that HIV-positive patients who exhibit symptoms of HIV infection are closely monitored for anemia so that they can receive timely treatment and education regarding anemia.

Unlike previous studies [1], [3], [21], [22], [23], we did not find a statistically significant difference in the overall prevalence of anemia in men and women. However, we found that moderate and severe anemia was more prevalent among women, while mild anemia was more prevalent among men. This is similar to the result of a previous study [52], in which the prevalence of anemia in HIV patients not receiving ART was higher in men than women but that marked anemia was more common in women.

Although previous studies have reported higher rates of anemia among patients on a zidovudine-containing ART regimen [3], [53], [54], our study did not find an association between ART regimen and anemia. Approximately 75% of our sample was on a zidovudine-containing regimen. With estimates of anemia incidence after zidovudine initiation ranging from 4% to 34.5% in low resources settings [55], [56], [57], [58], [59] and usually occurring between six months and one year of treatment initiation [55], [56], it is possible that participants in our study who experienced anemia after initiating zidovudine were promptly switched to a different ART regimen. Furthermore, in at least one previous study [3], prescription of zidovudine was positively associated with drug-related anemia but negatively associated with diagnosis of anemia unrelated to drugs. Our methods did not allow us to differentiate between drug-related anemia and anemia unrelated to drugs. Thus, the protective effect of zidovudine against anemia unrelated to drugs and the possibility of more careful monitoring and management of other causes of anemia among zidovudine recipients may have outweighed the risk of drug related anemia.

A major strength of this study was the utilization of a community-based sample. Many previous studies [22], [24], [25], [26] recruited participants from a hospital or tertiary care setting or at a specific stage of HIV-progression [2]. Our sample included participants at different stages of disease progression who have varying access to health care and ART.

Some limitations to our study should also be noted. First, we did not collect data from medical records, but rather relied on self-reported data. Participants' answers may also have been influenced by social desirability, although our methodology tried to minimize this effect by ensuring confidentiality. Second, recall bias may have caused some inaccuracies in our data. We tried to minimize this effect by using a 1-month recall for HIV symptoms and alcohol use and 6-month recall for injection drug use. Third, our participants were all members of HIV-related NGOs. Therefore, our results represent only the subpopulation of PLWHA who are associated with the network of HIV-related organizations in Kathmandu. Finally, our study design did not allow us to determine if participants were anemic prior to becoming infected with HIV. We recognize that with high rates of anemia in the general population, it is probable that some participants were anemic prior to becoming infected with HIV. However, all of our participants on ART must have been screened for anemia and treated, if necessary, during treatment initiation as suggested by the national ART guidelines [60]. Moreover, the rates of anemia in our participants (64.7% in women and 49.2% in men) are much higher than the most recent estimates of anemia in the general population (34% in non-pregnant women) in the country [35], suggesting an increased risk of anemia in this population.

Despite these limitations, our study revealed a high level of anemia among a community-based sample of HIV-positive people in the Kathmandu Valley, Nepal. Rates of anemia were highest among those who had the highest burden of HIV symptoms. In 2004 the Anemia in HIV Working Group developed a set of guidelines for monitoring and safely treating anemia in PLWHA. These guidelines stressed the importance of initiating ART when appropriate, routinely monitoring hemoglobin levels, addressing the correctable causes of anemia (such as malaria, hookworm, malnutrition), and initiating epoetin alfa therapy once correctable causes of anemia have been ruled out [61]. In line with these guidelines, Nepal's national guidelines [60] for HIV treatment suggest monitoring of anemia before ART initiation and at 2 weeks, 1-month, 2-months, and 3-months after ART initiation and then at every 3-months thereafter. Our results reinforce the importance of adhering to the national ART guidelines for monitoring anemia among PLWHA. Moreover, monitoring of anemia at HIV diagnosis and during pre-ART care would help early identification and treatment of appropriate cases. Patients should be counseled on their risk of developing anemia and on the importance of seeking timely treatment for their HIV symptoms and conditions such as hookworm infection to help reduce their risk of anemia. Finally, clinicians should treat anemia with epoetin alfa therapy as well as other conditions as necessary.
